# Rheological and Mechanical Characterization of Dual-Curing Thiol-Acrylate-Epoxy Thermosets for Advanced Applications

**DOI:** 10.3390/polym11060997

**Published:** 2019-06-04

**Authors:** Claudio Russo, Xavier Fernández-Francos, Silvia De la Flor

**Affiliations:** 1 Department of Mechanical Engineering, Universitat Rovira i Virgili, Av. Països Catalans 26, 43007 Tarragona, Spain; claudio.russo@urv.cat; 2 Thermodynamics Laboratory, ETSEIB, Universitat Politècnica de Catalunya, Av. Diagonal 647, 08028 Barcelona, Spain

**Keywords:** epoxy, thiol, acrylate, dual-curing, shape-memory polymer, adhesive

## Abstract

Mechanical and rheological properties of novel dual-curing system based on sequential thiol-acrylate and thiol-epoxy reactions are studied with the aim of addressing the obtained materials to suitable advanced applications. The crosslinking process is studied by rheological analysis in order to determine conversion at gelation and the critical ratio. These parameters are used to discuss the intermediate material structure for each acrylate proportion and their possible application in the context of dual-curing and multi-step processing scenarios. Results from dynamo-mechanical analysis and mechanical testing demonstrate the high versatility materials under investigation and revealed a wide range of achievable final properties by simply varying the proportion between acrylate and thiol group. The intermediate stability between curing stages has been analysed in terms of their thermal and mechanical properties, showing that these materials can be stored at different temperatures for a relevant amount of time without experiencing significant effects on the processability. Experimental tests were made to visually demonstrate the versatility of these materials. Qualitative tests on the obtained materials confirm the possibility of obtaining complex shaped samples and highlight interesting shape-memory and adhesive properties.

## 1. Introduction

Crosslinked polymeric materials are used in many application fields because of their excellent thermal and mechanical properties (i.e., aviation, automobile, structures or coatings) [1]. The formation of crosslinked thermosetting network is a non-reversible process involving drastic changes in polymer and network structures which leads to relevant restrictions in shape designs. Recently, the increasing need to reach complex shape designs to cater for the high demand of complex shaped smart materials (i.e., bio-inspired devices or shape-changing materials [2]), is focusing the attention on alternative curing techniques which allow to overcome thermosets limitations in shape. In this context, dual-curing polymer systems have attracted a growing interest as they represent a versatile approach for better controlling network formation and properties during processing [3,4].

Dual-curing processing is a promising methodology to develop thermosetting polymers taking advantage of two compatible and well-controlled crosslinking reactions [5]. These reactions can be simultaneously or sequentially triggered by different stimuli as well as difference in reaction kinetics. Sequential dual-curing allows to obtain materials with two different sets of properties after the first curing reaction (intermediate material) and second curing reaction (final material). This kind of processing is becoming attractive due to the possibility to attain complex three-dimensional structures by means of the accurate control of the materials properties in the intermediate state (i.e., low *T*_g_, low crosslinking density and high deformability).

Successful sequential dual-curing processing requires that: (i) both polymerization reactions are selective and compatible so that no undesired inhibition or reactivity effects take place; (ii) they can be triggered using different stimulus such as UV light [6] or temperature [7], or else they have sufficiently different reaction rates so that they can be controlled from a kinetics point of view; (iii) the properties of the final and intermediate stages can be custom-tailored changing the composition of the formulation.

Thermosetting dual-curing materials have been obtained from the combination of a large variety of reactions, but “Click” reactions represent one of the most effective tools to obtain such systems. “Click chemistry” defines a class of reactions that are highly efficient, orthogonal and selective [8]. Moreover, they reach high yields, they can react in mild or solvent-less conditions and they can be applied to broad range of compounds [9,10,11]. For those reasons, “click” reactions are widely used in thermosets preparation and are fit for combination in dual-curing procedures. In particular, thiol-click reactions have attracted great interest due to their advantages (high conversion, solvent-free, oxygen resistance, etc.) [11,12,13,14] which makes them suitable to prepare crosslinked polymers in a fast and efficient way. 

Michael-type addition reactions are currently used in dual-curing processing because of the variety of commercially available nucleophiles (Michael donors) and activated double bond compounds (Michael acceptors) that can be used in such processes. One of the most common and clickable Michael donors are thiols. Although thiol-acrylate addiction has a good combination of reactivity, versatility and cost, the final thermosetting structures exhibit week mechanical and thermal properties which makes them unable to fit the standards required for advanced applications [15]. On the other hand, thiol-epoxy “click” reaction, which is also used in dual-curing systems, leads to soft functional materials with enhanced mechanical properties such as high deformability, resistance at break, high impact resistance and adhesion. All these properties make them suitable for advanced applications as coatings [16], adhesives [17] and shape-memory actuators [18]. The combination of these reactions in dual-curing procedure is already reported in the literature: Konuray et al. [6] presented a novel photolatent dual-cure thiol-acrylate-epoxy system where the first curing reaction is the thiol-Michael reaction triggered by UV light (a photobase generator was used as catalyst), and the second reaction is a thiol-epoxy activated at higher temperature. Jin et al. [7] prepared thiol-epoxy-acrylate hybrid polymer networks combining nucleophilic thiol-acrylate Michael addition and thiol-epoxy reactions in a one-pot simultaneous dual-cure catalyzed by 1,8-diazabicyclo[5.4.0]undec-7-ene (DBU) at 80 °C. In our previous work [19] a new thiol-acrylate-epoxy sequential dual-curing system, where both reactions are activated using a single thermal catalyst, was developed. Although thiol-acrylate Michael addition and thiol-epoxy “click” reaction are both thermally-activated within a similar temperature range, the former’s faster reaction kinetic ensures the sequentiality of the process. 

Suitable choice of monomer feed ratio, structure and functionality makes it possible to obtain an intermediate thiol-acrylate conformable network after the first curing step. This network could be processed in complex shape designs and then, thanks to the presence of unreacted thiol and epoxy groups, a second crosslinking reaction could be triggered leading to the final materials. Therefore, complex-shaped thermosets can be achieved thanks to the two-step processing. Since thermosetting networks formed by thiol-acrylate reaction generally exhibit flexible structures, the addition of a further thiol-epoxy crosslinking process could enhance the final properties of the thermosets. Thermal and mechanical properties of intermediate and final thermosets can be easily tailored controlling the ratio between acrylate and thiol groups (*r*_a_). Therefore, it is possible to obtain intermediate materials with properties ranging from liquid-like to gelled solid-like covering a wide range of possible applications. The critical ratio (*r*_c_), defined as the lower *r*_a_ at which gelation occurs within the first crosslinking process, sets a boundary line between liquid and solid-like intermediate materials. Therefore, this parameter must be accurately evaluated to address each resulting material to the adequate application. Further analysis of the chemorheological behaviour of these dual formulations, taking into consideration the complex effect of temperature and curing progress on the viscosity during processing [20,21], would also be highly valuable in the simulation and optimization of other processing scenarios [22].

Such design capabilities can be exploited to produce multi-layer assemblies with controlled layer thickness and complex shape, with the purpose of creating complex-shape mechanical actuators [18], making use of the good wetting properties of intermediate lightly crosslinked or nearly gelled materials and the good adhesion obtained after the thiol-epoxy reaction. 

The aim of this work is to characterize the mechanical, thermal and rheological properties of a novel thiol-acrylate-epoxy dual-curing system. Mixtures at various thiol-acrylate ratios, covering the entire range between 0 and 1, were prepared and final thermosets properties were studied. The evolution of rheological properties during the curing process were monitored to determine the actual critical ratio for this system. After that, the main mechanical properties (hardness, flexural and tensile modulus, tensile strength and deformation at break), of the totally cured materials (final thermosets) were evaluated in order to highlight the high versatility of the applications of the dual-curing system developed. To characterize the storage time of the intermediate materials, the evaluation of the latency of the second curing reaction was carried out on two different formulations, one with a *r*_a_ > *r*_c_ and one with *r*_a_ < *r*_c_. The progression of the thiol-epoxy was evaluated in terms of residual heat (DSC), complex viscosity η* (rheometer) and tensile module *E*_t_. Lastly, qualitative and visual demonstrations of different applications are presented to show the possibility of this systems to be exploited for two-stage adhesive technologies and for the preparation of complex-shaped shape-memory actuators.

## 2. Materials and Methods

### 2.1. Materials

Diglycidyl ether of bisphenol A (DGEBA, EPIKOTE™ Resin 828, with an epoxy equivalent weight of 187 g/eq) was supplied by Hexion Specialty Chemicals (Columbus, OH, USA), and dried under vacuum at 80 °C before use. Pentaerythritol tetrakis(3-mercaptopropionate) (S4, 122.17 g/ee) and tricyclo[5.2.1.02,6]decanedimethanol diacrylate (TCDDA, 152.2 g/ee) were purchased from Sigma-Aldrich (St. Louis, MO, USA) and used as received. 4-(*N*,*N*-dimethylamino)pyridine (DMAP) was purchased from Fluka (Seelze, Germany)and used at 0.05 phr to act as catalyst for both curing reactions. Different formulations have been prepared varying the ratio between acrylate groups and thiol groups (*r*_a_). All samples were cured at 40 °C for 2 h (1st step), 80 °C for 3 h (2nd step) and 100 °C for 1 h (post curing).

Formulations have been coded as TCDDAxx where xx is the *r*_a_ (00 and 10 correspond to respectively thiol-epoxy and thiol-acrylate pure formulations). The compositions of the investigated formulations are listed in Table 1.

### 2.2. Characterization Techniques

#### 2.2.1. Rheological Characterization of the Gel Point and *r*_c_

The dual-curing process was monitored using a rheometer AR-G2 TA Instruments, (New Castle, DE, USA), equipped with an electrical heated plate device (EHP) and 20 mm parallel plate geometry. The evolution of the storage (*G*′) and loss modulus (*G*″) was monitored through dynamo-mechanical experiment at 30 °C for 8 h. The oscillation amplitude was set at 0.2% and the frequencies at 0.5, 1.75 and 3 Hz. Gel point was determined as the tanδ crossover at the three different frequencies and the first gelled formulation (*r*_c_) was defined as the formulation with the lower *r*_a_ that shows a gel point within the first curing process. Gelation during dual-curing procedure was also tested step-wise as follows: first curing stage 3 h at 30 °C with an amplitude of 3%; after that a temperature ramp from 30 to 60 °C at 2 °C/min (with the same oscillation amplitude) and finally second curing stage 2 h at 60 °C with an amplitude of 0.2%. Three different frequencies of 0.5, 1.75, 3 Hz were continuously measured during the whole procedure. The experimental *r*_c_ was compared with the theoretical value obtained for ideal step-wise processes using the Flory-Stockmayer theory, as follows:

(1) $$r_{c}=\frac{1}{\left(f_{\mathrm{acrylate}}-1\right)\cdot \left(f_{\mathrm{thiol}}-1\right)}$$

where *f*_acrylate_ and *f*_thiol_ are the average functionality of acrylate and thiol monomers. 

Complex viscosity (η*) of the intermediate materials were recorded as function of angular frequency ω (rad/s) at constant deformation in the range of linear viscoelasticity, obtained from constant shear elastic modulus (*G*′) in a strain sweep experiment at 1 Hz, always at 25 °C.

#### 2.2.2. Mechanical Properties

Final thermosets were analysed with a TA Instruments DMA Q800 (New Castle, DE, USA) equipped with 3-point bending clamp (15 mm) to characterize the relaxation process. Prismatic rectangular samples (15 × 6 × 2.5 mm^3^) were analysed in oscillation mode at 1 Hz, 0.1% of strain amplitude and imposing a temperature ramp of 3 °C/min from −20 to 120 °C. The *T*_g_ was determined as the tanδ peak temperature, glassy (*E*_g_) and rubbery (*E*_r_) moduli were determined at 0 and 100 °C, respectively.

Mechanical properties were tested at room temperature to investigate the processability of final materials at normal or usual operating temperatures.

The Flexural modulus (E) of final materials was determined with the same apparatus by means of a force ramp at a constant rate of 1 N/min in controlled-force mode. The slope (m) within the linear zone of force-displacement curve was obtained. *E* was calculated in accordance with the following equation:

(2) $$E=\frac{L^{3}m}{4wt^{3}}$$

where L is the support span and w and t are the width and the thickness of test sample respectively.

Tensile properties of dog bone shaped samples (80 mm × 25 mm × 1.5 mm) were obtained by tensile test on a Shimadzu AGS-X 10 kN (Kyoto, Japan) testing machine at 10 mm/min speed, according to ASTM D638-14 (ASTM International, West Conshohocken, PA, USA, 2014) standard. Shore hardness was measured with an Affri durometer type D (Shore-D hardness) according to ASTM D2240-15 (ASTM International, West Conshohocken, PA, USA, 2015) in samples of 4 mm thickness. Ten measurements were done in each sample and the average result is presented.

#### 2.2.3. Latency Test on Intermediate Materials

Latency tests were performed for TCDDA02 and TCDDA06 intermediate thermosets at two different storage conditions (5 and 22 °C). Samples at different storage times were tested using a differential scanning calorimeter (DSC) Mettler DSC-821e (Mettler-Toledo, Greifensee, Switzerland), calibrated using an In standard (heat flow calibration) and an In-Pb-Zn standard (T calibration). Samples of approximately 10 mg were placed in aluminium pans with pierced lids and cured in the oven. After the first curing stage, samples were stored in a climatic chamber at 5 and 22 °C and were periodically tested by DSC dynamic analysis from −20 to 200 °C with a heating rate of 10 °C/min under N_2_ atmosphere. The residual heat of the second curing step has been used as measure of the curing degree reached by the samples during the storage. 

The evolution of the intermediate materials properties, during a representative storage time interval, were monitored by measuring at *T*_room_ the tensile modulus for sample TCDDA06 and the viscosity for sample TCDDA02, since the latter leads to a liquid-like intermediate.

## 3. Results

### 3.1. Rheological Analysis

**Figure 1:**
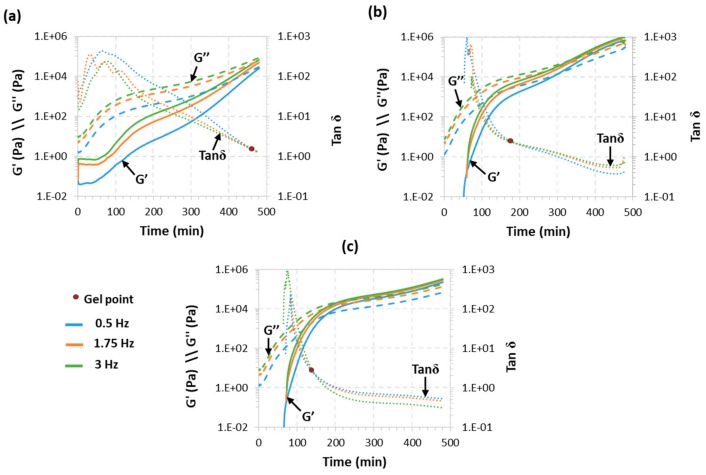
Evolution of storage modulus (*G*′), loss modulus (*G*″) and tanδ with time at 30 °C for samples: TCDDA04 (**a**); TCDDA045 (**b**) and TCDDA05 (**c**).

Rheological analysis is accepted as a reliable tool to determine the gelation of thermosetting systems. The critical ratio of our system was determined to be *r*_c_ = 0.33 using Equation (1). In light of the approximations made and taking into account previous experience [23], we expected the real critical ratio to be higher than the theoretical one, so we started the rheological analysis from *r*_a_ = 0.4. The curing process was monitored at an isothermal temperature of 30 °C according to the results obtained in our previous work [19]. Figure 1 shows the rheological monitoring of the isothermal curing process of three formulations with *r*_a_ values above the theoretical *r*_c_, TCDDA04, TCDDA045 and TCDDA05. As shown in these figures, the network formation during the polymerization process results in a drastic increase of both storage (*G*′) and loss (*G*′) moduli. Two polymerization processes are visible in all three figures since the increase in moduli shows a two-step trend. 

For all three samples thiol-acrylate reaction occurs within the first 180 min (as verified with DSC, results similar to those reported previously [19], not shown) leaves a reaction tale overlapped with the thiol-epoxy which proceed at a low rate because of the low curing temperature. Along the process, the material goes through a substantial transformation: at the beginning the material has liquid-like behaviour and *G*″ is higher than *G*′. As the reaction takes place, structure starts to develop leading to molecular weight increase, resulting in an increase of *G*″ and also *G*′. When gelation takes place, a network develops, and the material start to behave as solid (*G*′ > *G*″). Figure 1a shows that no gelation is detected in the first three hours of curing of formulation TCDDA04, and the gel point is clearly visible only after the second crosslinking process is started. Gelation occurs right before the end of the analysis and *G*″ remains higher than *G*′. Raising the *r*_a_ to 0.45, gelation occurs after 194 min (Figure 1b) and it seems to take place right after the first curing reaction ends. Finally, as shown in Figure 1c, TCDDA05 formulation has the gel point within the first curing process and during the first 3 h of curing. As a result, the *r*_a_ of 0.45–0.5 can be established as the actual *r*_c_. As already reported for other dual-curing systems [23], the effective critical ratio (*r*_c_ = 0.45–0.5) is higher than the one obtained theoretically from the Flory-Stockmayer relationship. Deviation from the ideal step-wise behaviour could be explained by the action of intramolecular cyclization which would lead to a delay in gelation phenomenon [24,25]. The presence of impurities in the S4 (reported by Sigma-Aldrich to be lower than 5%) could reduce the actual functionality of the curing agents resulting in higher conversion at gelation.

**Figure 2:**
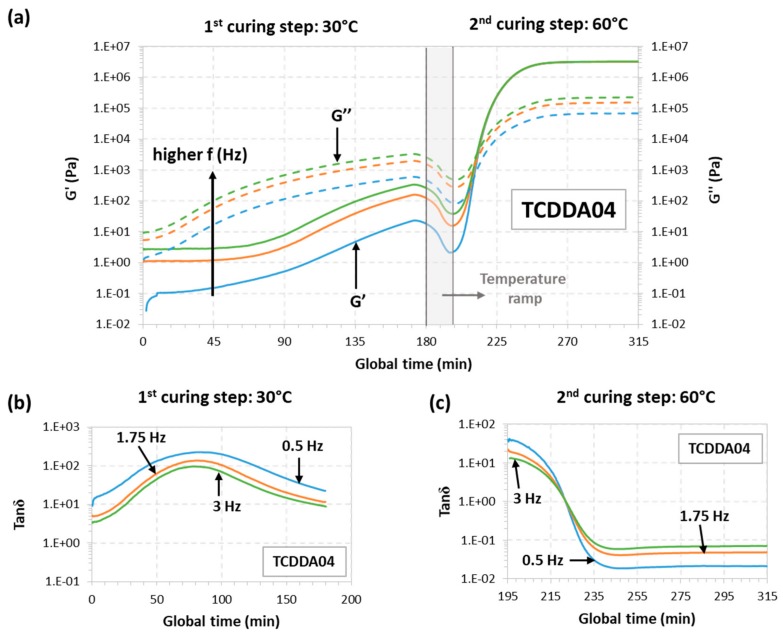
Rheological analysis of TCDDA04 dual-curing procedure: (**a**) Storage modulus (*G*′) and loss modulus (*G*″) evolution with time during the whole dual-curing process; (**b**) evolution of tanδ with time during the 1st curing stage and (**c**) evolution of tanδ during the 2nd curing stage.

**Figure 3:**
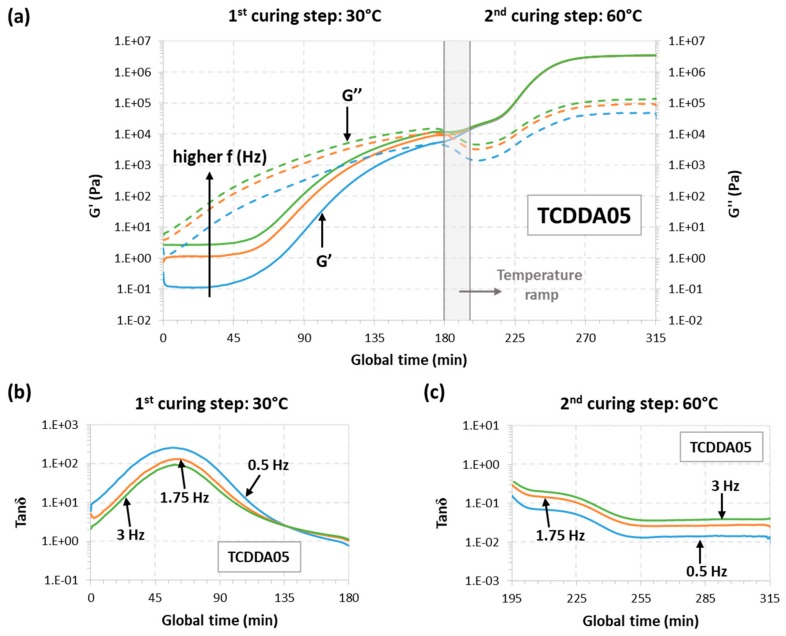
Rheological analysis of TCDDA05 dual-curing procedure: (**a**) Storage modulus (*G*′) and loss modulus (*G*″) evolution with time during the whole dual-curing process; (**b**) evolution of tanδ with time during the 1st curing stage and (**c**) evolution of tanδ during the 2nd curing stage.

The rheological behaviour for samples TCDD04 and TCDDA05 was also monitored during a dual-curing procedure divided as follows: a first isothermal step at 30 °C for 180 min a temperature ramp at 2 °C/min from 30 to 60 °C and, finally, a second isothermal step at 60 °C until *G*′ and *G*″ reach a plateau. The results can be observed in Figure 2 (TCDD04) and Figure 3 (TCDDA05). 

Starting with TCDDA04, Figure 2a shows that, *G*″ remains higher than *G*′ along the first curing stage thus, the material is still acting as liquid since the network is not already formed. *G*″ decreases during the heating ramp due to the decrease in viscosity with temperature, and so does *G*′. Crossover of *G*′ and *G*″ is clearly visible in the second stage, within the thiol-epoxy crosslinking process. The evolution of tanδ during the first and the second stage of curing is presented, respectively, in Figure 2b,c. As expected, tanδ crossover is only visible about 30 min after the second stage has started. In the case of formulation TCDDA05, Figure 3a shows that, for crossover of *G*′ and *G*″ takes place at the very end of the first curing stage, but the tanδ crossover is clearly observed in the first curing step (Figure 3b), therefore confirming that an intermediate gelled material could be obtained with a proportion of 0.5. It can also be observed in Figure 3a a slight decrease in *G*″ during the heating ramp from 30 to 60 °C, while *G*′ remains fairly constant and even increases due to the existence of an incipient network that is developing. 

### 3.2. Mechanical and Thermomechanical Analysis

**Figure 4:**
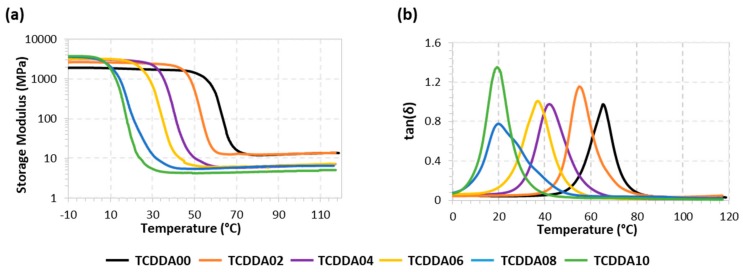
Storage Modulus (**a**) and tanδ (**b**) versus temperature during dynamic mechanical analysis.

In this section, thermomechanical properties and the mechanical behaviour of the final thermosets are discussed. The aim of this characterization is to evaluate the properties which determine the final use of the material in real applications. Figure 4 presents the evolution of storage modulus *E*′ and tanδ with temperature during the network relaxation of the different materials, obtained by a DMA temperature sweep analysis. Table 2 summarizes some relevant parameters associated with the network relaxation. As expected, raising the proportion between thiol and acrylate results in lower *T*_g_s for the final materials because of the softening effect of the thiol-acrylate network. The shape of the tanδ peak during the material relaxation can be correlated with its network structure: the higher and narrower the peak of tanδ, the more homogeneous and mobile the network structure [26]. Similar values of FWHM around 11 °C were obtained with both thiol-epoxy and thiol-acrylate pure formulation since these networks were built with the same crosslinking functionality. Dual formulations present broader tanδ transition paths due to the higher heterogeneity of the network, which is made up of two different crosslinking reactions. Belmonte at al. [27] reported a strong relationship between the sharpness of the relaxation process and the rate of the shape-recovery process. Materials with higher FWHM values are unfavourable for shape-memory applications because broader relaxation profile leads to a slowdown of the recovering process. In general, broad transitions lead to an undesired anticipation of the softening of the materials with respect to the expected *T*_g_. It can also be observed that the relaxed modulus *E*′_r_ decreases with increasing acrylate ratio, as a consequence of the higher mobility of the TCDDA network in contrast with the DGEBA network, rather than a difference in crosslinking density. In contrast, the glassy modulus *E*′_g_ increased with increasing acrylate ratio, suggesting that a better chain packing and stronger intermolecular interactions take place in acrylate rich formulations. Tentatively, this can be rationalized in terms of the higher mobility of the TCDDA monomer in comparison with DGEBA, and the higher presence of polar carbonyl groups. Flexural Modulus (E) for all the samples was calculated from 3-point-bending test by means of Equation (2). As can be observed in Table 2, some samples present a very low value of E because their relaxation processes lie around room temperature (see Figure 4). Although these results are not representative values of Young Moduli in glassy state for these samples, they provide an effective measure of the material behaviour at the temperature of usage. Low flexural moduli result in high conformability at room temperature making these materials suitable for advanced applications based on soft materials (i.e., soft robotics).

**Figure 5:**
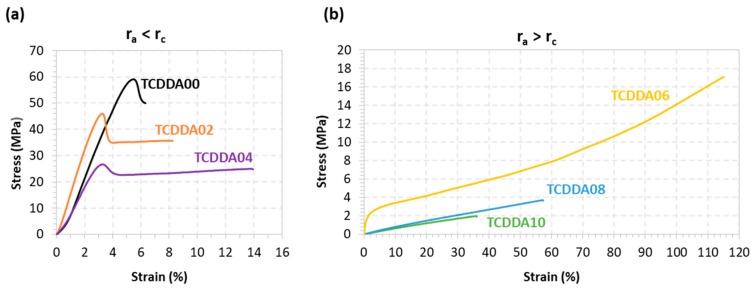
Stress-strain curves from tensile tests on final materials: (**a**) *r*_a_ < *r*_c_; (**b**) *r*_a_ > *r*_c_.

Tensile behaviour of final materials was also evaluated from tensile test in a universal testing machine. Tests were made at room temperature and the data obtained is presented in Table 3. Significant differences in terms of tensile modulus are observed between the thermosets above and below the *r*_c_. This trend is due to the shift of the glass transition towards *T*_room_ when increasing the acrylate proportion, with the consequent softening of the materials. The effect of the acrylate proportion is clearly visible in the stress-strain curves in Figure 5. When *r*_a_ = 0 (sample TCDDA00), a sudden fracture appears right after the yielding point and almost no plastic deformation is observed. When the acrylate ratio *r*_a_ is raised, a gradual increase in network deformability is observed in samples TCDDA02 and TCDDA04 (Figure 5a). A drastic change in tensile behaviour at *T*_room_ is observed in samples with *r*_a_ > *r*_c_ (Figure 5b). For TCDDA06 (*r*_a_ = 0.6), *T*_g_ ≈ *T*_room_ and network relaxation occurs during the strain-stress experiment. Network relaxation allows a higher dissipation of stress by viscous friction of polymer chains, reaching higher deformability and a relative high strength value at break with strain hardening at the end, as commonly observed in the programming of shape-memory thermosets at temperatures close to their relaxation temperature. However, when *r*_a_ > 0.6 (samples TCDDA08 and TCDDA10), it is observed that *T*_g_ < *T*_room_. In consequence, this stress-absorbing network relaxation mechanism is no longer operative, and therefore it is the relaxed network structure the main responsible for the mechanical response. A low elastic modulus is measured and, given the limited stretching ability of the polymer chains, a low stress at break σ_max_ is obtained. Consequently, increasing the epoxy-thiol content in the final network, higher strength at break can be obtained. The same effect is also visible in terms of strain energy density as it can be appreciated in Table 3. The differences in network relaxation state at *T*_room_ results in an increase of the material capability to absorb energy during deformation which achieves the highest value for TCDDA06 and then drastically decrease for TCDDA08 and TCDDA10. A similar trend is observed in hardness measurements; hence the same conclusions are applicable to this property. From the analysis of the data reported in Table 3 and Figure 5, it can be observed that the behaviour of TCDDA02 and TCDDA08 is very close to that of TCDDA00 and TCDDA10, respectively, suggesting that small deviations from the pure networks produce minor effects on final thermosets properties.

This analysis confirms that a wide range of final properties can be obtained by varying the composition of the formulation: the thiol-acrylate proportion in the network has been proved to have a softening effect on the final network, resulting in lower *T*_g_ and higher capability of absorbing energy during application deformation process. An interesting combination of these two reactions is present in the 0.6 proportion between acrylate and thiol: a highly deformable final material is be obtained together with an intermediate gelled network that make it suitable for application in which two-step processing is required (with a highly conformable manufacturing step in the intermediate one). On the other hand, acrylate proportions between 0.2 and 0.4 lead to a decrease in *T*_g_ with respect to the characteristic thiol-epoxy *T*_g_, without a major loss in terms of mechanical properties (final strength, *E*_t_, hardness). In this case, these materials could be exploited for two-stage application in which a viscous intermediate is required, such as adhesives or coatings.

### 3.3. Latency and Storage Time

The evaluation of latency of the second reaction represents a crucial analysis in dual-curing procedure. The storage time of the intermediate materials is strictly related to the amount of time required to activate the second reaction at *T*_room_ (or at different storage temperature), without significant alteration of the intermediate material properties. For that purpose, we chose to analyse the storage stability of TCDDA02 and TCDDA06 formulations.

**Figure 6:**
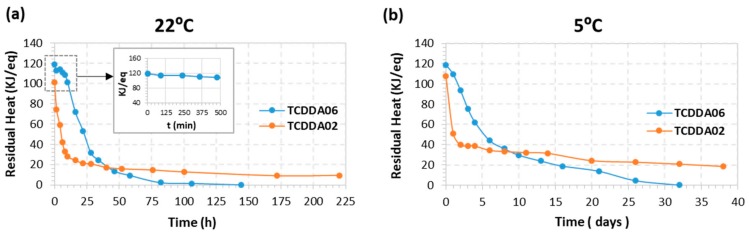
Results of latency evaluation at two different storage temperature for TCDDA02 and TCDDA06 formulations.

To begin with, the first curing stage was carried out in the oven at 40 °C for 2 h. After that, they were stored at 22 and 5 °C in a controlled climatic chamber. The samples were analysed by DSC at determined storage times in order to monitor the evolution of residual heat with storage. As shown in Figure 6a, intermediate TCDDA06 material experiences no significant changes in residual heat during 10 hours of storage at 22 °C. After that, a drastic decrease in residual heat is observed and the samples take six days (144 h) to react completely curing at storage conditions. On the contrary, TCDDA02 shows a rapid decrease in residual heat during the first six hours of storage and it reaches a plateau after 24 h. In this case, even if the thiol-epoxy reaction proceeds faster thus considerably reducing the storage time, it never reaches the complete curing because vitrification takes place during storage. Main differences between TCDDA02 and TCDDA06 can be explained by the higher epoxy content of TCDDA02, which enhances the reactivity of the epoxy-thiol reaction [28] and is therefore less stable. In fact, premature activation of the epoxy-thiol reaction might also take place during the first curing step. A practical consequence of this is that controlled curing sequences are more easily achieved in dual formulations with a moderate or low epoxy content. In addition, the fully cured TCDDA02 material has a higher *T*_g_, clearly above storage temperature (see Table 2), therefore explaining vitrification during storage. In contrast, the lower of *T*_g_ of TCDDA06 (see Table 2, calorimetric *T*_g_ is even lower [19]) makes it possible to react completely under storage conditions.

The same effects are observed when the samples are stored at 5 °C (Figure 6b): after 1–2 days of stability, TCDDA06 shows a gradual progression of the second reaction until no residual heat is registered, while TCDDA02 reach the final plateau after only 2 days of storage. 

**Figure 7:**
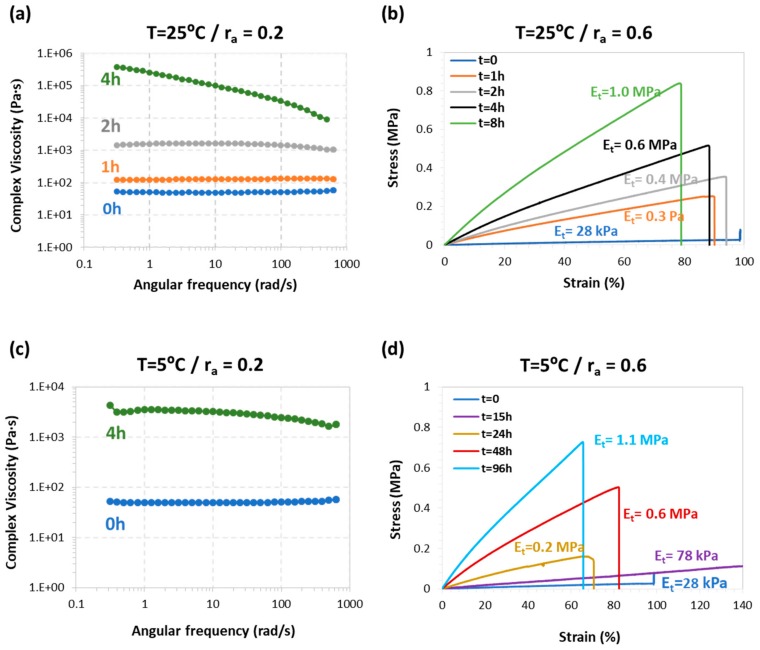
Results of the mechanical evaluation of the second reaction latency: (**a**) changes in complex viscosity of TCDDA02 during 25 °C storage; (**b**) evolution of the tensile modulus of TCDDA06 intermediate at 25 °C storage temperature; (**c**) changes in complex viscosity of TCDDA02 during 5 °C storage and (**d**) evolution of the tensile modulus of TCDDA06 intermediate at 5 °C storage temperature.

Furthermore, we studied the effect of storage on the intermediate mechanical in order to assess how the workability during the intermediate step is affected by storage time and conditions. Since TCDDA02 has a liquid-like intermediate stage, the increase of complex viscosity during storage was used to evaluate latency. During 4 hours at 25 °C (Figure 7a) a significant increase from 50 Pa·s to value of about 300 kPa·s (both measured at 1 Hz and at 25 °C) can be observed. When the samples were stored at 5 °C, an increase up to 3–4 kPa·s (measured at 1 Hz and at 25 °C) is observed (Figure 7c), two orders of magnitude less with respect the storage at 25 °C. Although this increase in complex viscosity could affect the processing ability of these materials, there is enough time to process the material in the intermediate state for two-stage applications. Tensile tests were performed on intermediate TCDDA06 materials in order to evaluate the increase of the tensile modulus during the storage process. As shown in Figure 7b, the initial modulus E_t_ has a value of 28 kPa, and it increases gradually up to 1 MPa after eight hours of storage at 25 °C. However, storage at 5 °C delays significantly the evolution of the modulus, producing the same increase up to 1.1 MPa in about four days (Figure 7d) confirming that these intermediate materials can be stored for an appreciable time without affecting the processability in the intermediate stage. 

### 3.4. Stage Processing Application

#### 3.4.1. Adhesive Bonding

**Figure 8:**
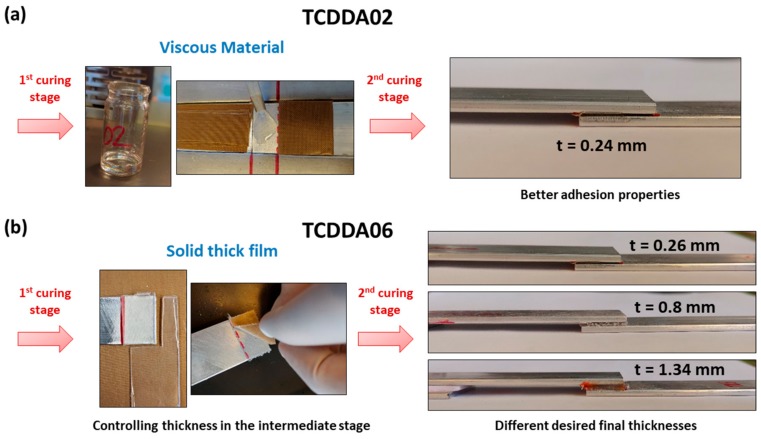
Processing of the adhesive bonding: (**a**) adhesive bonding with the viscous intermediate TCDDA02 material; (**b**) adhesive bonding with the solid intermediate film of TCDDA06.

The characteristic two-stage manufacturing process achievable with dual-curing system can be exploited for applications such as dry bonding adhesives [29]. Intermediate materials with *r*_a_ = 0.2 can be suitable to be used as viscous adhesive which can be easily spread on the adhesion surface. After the application, the second curing stage can be performed resulting in an extremely good final adhesion of the surfaces (Figure 8a). The curing temperature (and time) can be adjusted to the thermomechanical properties of the materials that we want to adhere. In this case, we can take advantage of the dual-curing procedure to reduce the shrinkage, but difficulties in final thickness control arise with thicker adhesive layers because of the viscosity in the intermediate stage. Using TCDDA06 formulation, intermediate solid materials sheets with different thickness can be obtained and used as solid-like adhesives that can be easily adjusted to the shape of the adhesion surface. As shown in Figure 8b, a precise control of the final thickness is thus achieved thanks to the gelled network formed during the first curing stage. The final thickness of the layer remains close to the designed thickness (the thickness of the mould used to prepare each sheet). The effective strength of the bonding and the relation between strength and adhesive layer thickness will be analysed in depth in a future work.

**Figure 9:**
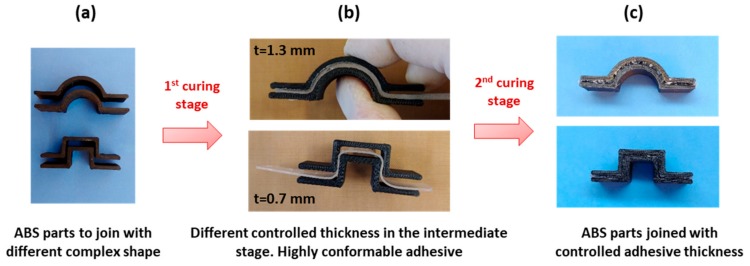
(**a**) Complex ABS (Acrylonitrile Butadiene Styrene) samples to join; (**b**) positioning the intermediate TCDDA06 film in-between the ABS samples to adhere and (**c**) result for the two different parts adhered.

The high conformability of the intermediate material with *r*_a_ = 0.6 can also be exploited for different kind of adhesion joint such as external joining of tubes or pipes [19]. The precise control of the thickness also results in the possibility of stick together different shapes, adjusting the thickness to the complexity of the shapes. In Figure 9, different thickness intermediate films of TCDDA06 were placed in-between two ABS pieces in order to stick them together in a one-piece-sample. 

#### 3.4.2. Shape-Memory Devices

**Figure 10:**
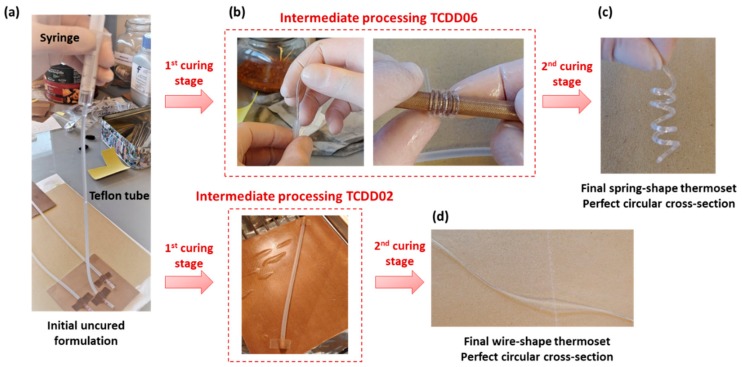
Preparation of a spring-shaped (**c**) and wire-shaped (**d**) samples from, respectively, TCDDA06 and TCDDA02 formulations (**a**,**b**).

Finally, the shape-memory behaviour of these materials has been qualitatively investigated taking advantage of the ease of processing of the intermediate material into complex shapes. Final spring-shaped samples were obtained with a two-step curing process of TCDDA06. The uncured formulation was poured into a thin silicon tube with the aid of a syringe (Figure 10a) and both ends of the tube were sealed. Afterwards it was cured through the first curing stage, with the tube acting as a mould, and intermediate materials in the form of wires were obtained after removing the silicon tube. The wires were wrapped around a cylindrical rod (Figure 10b) and cured for the second stage. As shown in Figure 10c, finished springs of controlled dimensions (perfect circular cross-section) were obtained with this procedure highlighting the capability of this material of being processed in complex shapes in the intermediate state. TCDDA02 wires were prepared removing the material from the silicon tube after the second curing step. Here again, excellent surface finish is obtained, with a constant cross-section in the wire along its whole length, as seen in Figure 10d.

**Figure 11:**
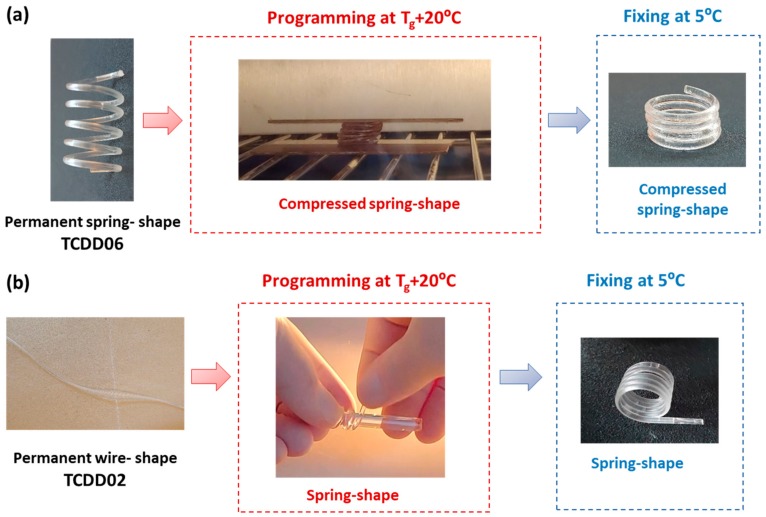
Programming of TCDDA06 and TCDDA02 samples: (**a**) TCDDA06 with a permanent spring shape is programmed in the form of a compressed spring. (**b**) TCDDA02 with a permanent shape of wire is rolled up around a cylindrical rod and a temporary spring shape is obtained.

The shape-memory properties of the spring and the wire shapes obtained were qualitatively tested. Springs prepared with TCDDA06 formulations were programmed at *T*_g_ + 20 °C in a compressed spring (Figure 11a). Conversely, TCDDA02 wire was programmed in the shape of spring rolling up the heated sample around a cylindrical rod (Figure 11b).

**Figure 12:**
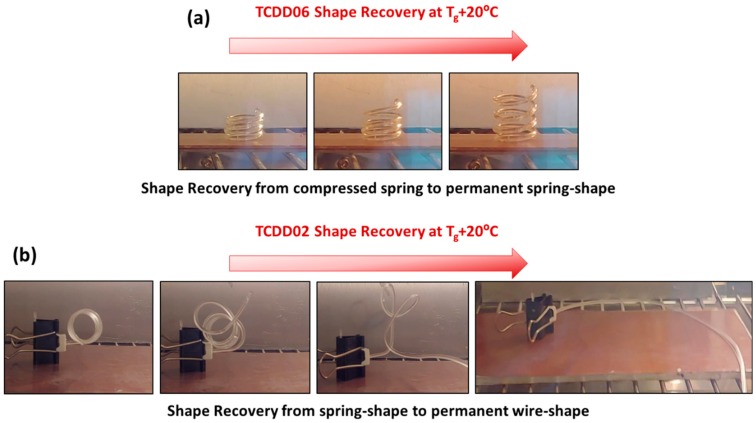
(**a**) TCDDA06 shape-recovery process to permanent spring shape (**b**) TCDDA02 shape-recovery process to permanent wire-shape.

Shape fixation was obtained in both situations cooling the deformed samples down to 5 °C. The permanent shape can be recovered in the oven by heating to 75 °C for TCDDA02 and 60 °C for TCDDA06 (*T*_g_ + 20 °C) as shown in Figure 12, leading to a complete recovering of the initial shape. As can be seen in Figure 12a the compressed spring recovered perfectly the initial length and the actuation seemed perfect to be exploited for a shape-memory actuator. However, the capability to give a work-output during the recovery process is somehow limited by the low stiffness of the final material. Some partially constrained recovery tests were done but the compressed spring sample was only able to recover is initial shape if a light weight was applied. This problem could be overcome by increasing the functionality of the epoxy resin thus obtaining final materials with tailorable stiffness and without affecting the high deformability of the intermediate state. This problem will be object of further studies.

## 4. Conclusions

In this work dual-curing thiol-acrylate-epoxy system was rheological and mechanically characterized and potential applications of the obtained materials were proposed. Rheological analysis was performed to determine the actual critical ratio for this system which defines the material behaviour in the intermediate stage. Mechanical and thermomechanical characterization of the materials resulting from the complete curing was performed by means of dynamo-mechanical analysis, Flexural 3-point-bending, tensile test and Shore-D hardness. Stability of the intermediate materials during storage were also evaluated by monitoring the advancement of the reaction in terms of thermal and mechanical properties. 

Rheological analysis of the curing process showed that gelation takes place within the first curing stage when the acrylate-thiol ratio is *r*_a_ > 0.45–0.5. A wide range of both solid-like and liquid-like intermediate materials can be obtained varying the *r*_a_ respectively above and below the *r*_c_. Thermomechanical characterization shows a network softening effect (decrease in flexural and *E*′_r_ moduli) together with a toughening effect in the glassy state (*E*′_g_) with the increase of *r*_a_.

Characterization of final thermoset mechanical behaviour at room temperature reveals that a wide range of properties can be obtained varying the proportion between acrylate and thiols group. An interesting combination of the properties of the two network is obtained with 0.6 proportion: final materials with high deformation at break with a gelled intermediate state is obtained. Moreover, slight variation from the pure thiol-epoxy mechanical properties were observed when a small thiol-acrylate proportion is added to the network, meaning that a two-stage curing procedure can be obtained without significant worsening the final materials properties. These evidenced features make this system suitable to be exploited for a large variety of advanced application such as shape-memory actuator and two-stage adhesives. 

Storage stability of the intermediate material, between the first and second curing stage, was investigated at storage temperatures of 25 and 5 °C and analysed with DSC and mechanical or rheological characterization. Differences in storage stability were found to be strictly related to the acrylate/epoxy proportion in the mixture. Formulations with a lower acrylate content have lower intermediate stability because the higher content of epoxy groups enhances the reactivity of the second stage thiol-epoxy reaction. Anyway, a relevant amount of time, in which processability of the materials was not affected, is observed for both formulations.

Visual qualitative examples have been presented to demonstrate the possibility to obtain complex shapes for shape-memory actuators and two-stage adhesives with controlled adhesive layer thickness and excellent adhesion.

## Figures and Tables

**Table 1:** Compositions of the different formulation of study.

Formulation	*r*_a_	Thiol (wt %)	DGEBA (wt %)	Acrylate (wt %)
TCDDA00	0	40	60	0
TCDDA02	0.2	40	49	0
TCDDA04	0.4	41	38	21
TCDDA045	0.45	42	35	23
TCDDA05	0.5	42	32	26
TCDDA06	0.6	42	26	32
TCDDA08	0.8	43	13	43
TCDDA10	1	45	0	55

**Table 2:** Thermomechanical data collected from DMA (dynamic mechanical analysis). Coefficients of variation less than 2% for thermomechanical data and 5% for Flexural Modulus.

Sample	*T*_g_ (°C)	*E*′_g_ (MPa)	*E*′_r_ (MPa)	FWHM (°C)	E (MPa) ^(a)^
TCDDA10	19.2	3679	4.85	11.3	5.23
TCDDA08	20.6	3422	6.43	18.3	5.76
TCDDA06	37.1	3230	5.82	13.6	21.83
TCDDA04	42.4	3181	6.50	14.3	39.82
TCDDA02	55.7	2648	13.06	11.4	1358
TCDDA00	65.5	1911	13.03	11.0	1028

^(a)^ calculated using Equation (2).

**Table 3:** Characteristic mechanical parameters obtained from tensile test and hardness measurements. Coefficients of variation less than 10% for stress, strain and tensile modulus, and less than 5% for Shore-D hardness.

	Formulations	*E*_t_ (MPa)	σ_max_ (MPa)	ε_max_ (%)	σ_break_ (MPa)	ε_break_ (%)	Strain Energy Density (KJ/m^3^)	Hardness (Shore-D)
*r*_a_ < *r*_c_	TCDDA00	1580.8	41.7	3.66	34.1	6.88	1861.7	72.5
	TCDDA02	1580.0	39.4	2.79	23.6	6.57	2299.5	73.1
	TCDDA04	1418.6	20.5	9.56	17.0	23.68	3531.9	65.3
*r*_a_ > *r*_c_	TCDDA06	372.8	17.0	108.94	17.0	109.04	9534.2	43.7
	TCDDA08	8.0	2.8	44.39	2.8	44.65	725.3	28.1
	TCDDA10	6.6	1.6	28.46	1.6	29.57	271.2	27.3
